# Emerging Roles of Sonic Hedgehog in Adult Neurological Diseases: Neurogenesis and Beyond

**DOI:** 10.3390/ijms19082423

**Published:** 2018-08-16

**Authors:** Shang-Der Chen, Jenq-Lin Yang, Wei-Chao Hwang, Ding-I Yang

**Affiliations:** 1 Department of Neurology, Kaohsiung Chang Gung Memorial Hospital, Kaohsiung City 83301, Taiwan; chensd@adm.cgmh.org.tw; 2 Institute for Translational Research in Biomedicine, Kaohsiung Chang Gung Memorial Hospital, Kaohsiung City 83301, Taiwan; jyang@adm.cgmh.org.tw; 3 College of Medicine, Chang Gung University, Taoyuan City 33302, Taiwan; 4 Department of Neurology, Taipei City Hospital, Taipei 11556, Taiwan; huangshiaowei@gmail.com; 5 Institute of Brain Science, National Yang-Ming University, Taipei 11221, Taiwan; 6 Brain Research Center, National Yang-Ming University, Taipei 11221, Taiwan

**Keywords:** sonic hedgehog, neurogenesis, anti-oxidation, anti-inflammation, autophagy, neurodegenerative diseases

## Abstract

Sonic hedgehog (Shh), a member of the hedgehog (Hh) family, was originally recognized as a morphogen possessing critical characters for neural development during embryogenesis. Recently, however, Shh has emerged as an important modulator in adult neural tissues through different mechanisms such as neurogenesis, anti-oxidation, anti-inflammation, and autophagy. Therefore, Shh may potentially have clinical application in neurodegenerative diseases and brain injuries. In this article, we present some examples, including ours, to show different aspects of Shh signaling and how Shh agonists or mimetics are used to alter the neuronal fates in various disease models, both in vitro and in vivo. Other potential mechanisms that are discussed include alteration of mitochondrial function and anti-aging effect; both are critical for age-related neurodegenerative diseases. A thorough understanding of the protective mechanisms elicited by Shh may provide a rationale to design innovative therapeutic regimens for various neurodegenerative diseases.

## 1. Introduction

The hedgehog gene (*Hh*) was first discovered in the fruit fly *Drosophila melanogaster* by Christiane Nüsslein-Volhard and Eric Wieschaus, two Nobel Laureates who were devoted to studying the genetic control of early embryonic development [1]. The loss of function mutation of *Hh* results in small pointed projections covering the larvae that are similar to the spikes of a *hedgehog* (*Hh*), hence the name. In mammals, there are three *Hh* family members, namely *sonic hedgehog* (*Shh*), *indian hedgehog* (*Ihh*), and *desert hedgehog* (*Dhh*). Among them, Shh is the best-studied ligand of the hedgehog signaling pathway. Shh is a soluble extracellular protein that was originally discovered to carry a function in cellular differentiation in the neural tube and limb bud with the growth of digits [2].

It was later found that Shh signaling is critical to regulating a variety of developmental processes in the nervous system, such as differentiation of ventral forebrain neurons and midbrain dopaminergic neurons as well as proliferation and differentiation of cerebellar neuronal precursors [3,4,5]. In addition to the developmental specification of the cell fate, Shh signaling plays crucial roles in early patterning of the embryonic brain, notably to regulate polarity of the central nervous system (CNS) [6] as well as to guide the ventral patterning in the spinal cord [7]. These features are important for craniofacial development because disruption of Shh signaling pathway may cause craniofacial neural crest cell death, thereby resulting in craniofacial anomalies in both vertebrate models and human populations [8,9]. During early stages of head formation, Shh is produced in three key domains, namely neuroectoderm of the ventral forebrain, facial ectoderm, and the pharyngeal endoderm [8,9]. Shh signaling is critical for orchestrating the fundamental organization of the craniofacial region [10,11]. Deletion of Shh in mice results in major craniofacial defects, such as alobar holoprosencephaly, cyclopia [12], stomodeum [13], or hypoplasia in the first pharyngeal arch [14,15].

Hh signaling is also important for tumor formation. Aberrant activation of Hh pathway during adult life can lead to tumorigenesis in both basal cell carcinoma [16] and medulloblastoma [17]. Furthermore, improper activation of this pathway has been shown in a variety of other types of human cancers, such as those in the brain, breast, gastrointestinal system, lung, and prostate. Paracrine effect of Hh secretion from the tumor to the surrounding stroma can advance tumorigenesis. This pathway also regulates proliferation of cancer stem cells and enhances tumor invasiveness. The topics of Hh pathway in tumor formation and the related clinical application for cancer treatment have been well reviewed elsewhere [18,19,20] and will not be further discussed in this article.

**Figure 1:**
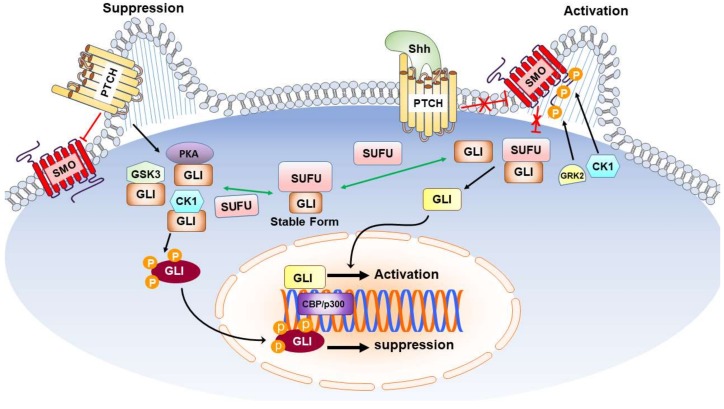
Sonic Hedgehog Signal Transduction Pathways. Shh acts on PTCH to relieve the inhibitory of PTCH on SMO, thereby activating the downstream pathway of SMO via binding with COS2. Upon binding of SMO to COS, Fu is released from COS2 complex and the freed Fu binds to SUFU to release GLI from SUFU complex. The freed GLI then enters the nucleus and, along with other transcription factors such as CBP/p300, binds to the promoters of target genes to regulate their expression. Black arrows indicate promotion or increasing; red lines with a “T” and/or “X” indicate inhibition, suppression, or decreasing; green arrows indicate competition. Abbreviation: Shh: Sonic Hedgehog; PTCH: Patched; SMO: Smoothened; CBP: CREB-binding protein; COS2: Costal-2; Fu: protein Fused; SUFU: Suppressor of Fused.

It is now generally accepted that the mammalian Hh signaling relies on the primary cilium, a microtubule-based protrusion with an antenna-like structure in the plasma membrane [21]. Shh, a secreted glycoprotein, can bind to a plasma membrane receptor called Patched (Ptch). In the absence of Shh, Ptch maintains the transmembrane transducer Smoothened (Smo) in an inactive state and allows the transcription factor glioma-associated oncogene homolog (Gli) to be phosphorylated by casein kinase 1 (CK1), glycogen synthase kinase-3 (GSK3), and protein kinase A (PKA) [22]. These phosphorylation events lead to proteolytic cleavage of the full-length Gli into Gli repressor, which suppresses the expression of target genes and hence inactivates the Hh signaling pathway. On the contrary, in the presence of Shh, the inactivated Ptch relieves its suppression on Smo, which is then phosphorylated by CK1 and G protein-coupled receptor kinase 2 (GRK2) [23]. The inhibitory activity of suppressor of Fused (SUFU) on Gli is then relieved with subsequent activation of signal transduction pathways to induce the transcription of target genes [24,25] such as N-Myc [26], Bcl-2 [27,28], and Bmi1 [29] that participate, respectively, in the regulation of proliferation, survival, and self-renewal. A schematic overview of Hh signaling pathway is shown in Figure 1.

Robust neurogenesis in the cerebral cortex during the embryonic stage is significantly influenced by Shh, which possesses a pleiotropic effect to the developing CNS and directs neural cells into proliferation, specification, as well as growth of axons and dendrites in various CNS regions that include forebrain, hindbrain, and spinal cord. Shh also functions as a mitogen to regulate proliferation and survival of neural stem cells (NSCs)/neural progenitor cells (NPCs) [30]. In addition to its notable importance at the beginning of life, Shh plays a vital role in regulating proliferation of NPCs in adult hippocampus [31,32]. Decreased expression of Shh is associated with senescence, thereby rendering the body more susceptible to aging-related disorders [33]. Accordingly, an active Hh signaling is particularly crucial for maintaining the activity of neurons in adults [34]. Consistent with this notion, Ptch and Smo, the Shh receptors, are expressed in the adult hippocampus and in the NPCs derived from hippocampus [31,35].

Shh can regulate the response of the mature brain to various types of damages such as ischemic insult, brain injury, and neurodegeneration [36,37,38]. Shh signaling may also enhance or reduce the extent of reactive astrogliosis, depending on the time interval, injury severity, and the inflammatory response of an insult [30,37,39,40,41].

Several studies, including ours, reveal that manipulation of the Shh pathway carries therapeutic potential in neurodegenerative disorders and cerebral ischemia [42,43,44,45,46,47,48]. We have reported before that Shh mediates brain-derived neurotrophic factor (BDNF)-induced neuroprotection against 3-nitropropionic acid (3-NP), which is an irreversible inhibitor of mitochondrial succinate dehydrogenase, also known as Complex II in the electron respiratory chain, that has been used to investigate the molecular mechanisms concerning cell death, mitochondrial dysfunction, and neurodegeneration in Huntington’s disease (HD) [47,49]. BDNF-mediated protection against 3-NP neurotoxicity was abolished by cyclopamine, the Shh pathway inhibitor. These results indicate that BDNF induces expression of Shh to mediate the beneficial effects against 3-NP neurotoxicity in rat cortical neurons [47]. We further validated that BDNF-dependent Shh expression and 3-NP resistance entail preceding induction of erythropoietin (EPO), thus verifying a signaling cascade of “BDNF → EPO → Shh → 3-NP resistance” in rat cortical neurons [46]. Recently, beneficial actions of Shh in ischemic injury have been noted [50,51,52]. By topical application of N-terminal fragment of Shh (Shh-N) and/or its specific inhibitor cyclopamine in fibrin glue over the peri-infarct cortex in the rat model of middle cerebral artery occlusion (MCAO), which mimics cerebral ischemia, we showed that Shh-N can attenuate protein oxidation and lipid peroxidation as well as increase neurogenesis and angiogenesis while decreasing astrocytosis [45]. It has also been demonstrated that, in hippocampal neurons, activation of the Shh signaling pathway may affect several features of mitochondrial function, such as increasing mitochondrial mass, inhibiting mitochondrial fission protein Drp1, and reducing mitochondrial fission while promoting mitochondrial elongation [53]. Shh can protect neurons against various stresses, including the amyloid β-peptide, high levels of glutamate, hydrogen peroxide, and rotenone, all are molecules or mediators related to ischemic injury or neurodegenerative diseases such as Alzheimer’s disease (AD) and Parkinson’s disease (PD) [53]. It is suggested that Shh may function as an anti-aging signal [33,54] based on several lines of evidence; these include the observations that the total Shh activity or inducibility are notably reduced in older animals [55], the age-related process of cellular senescence is blocked by active Shh signaling [56], and Shh-targeting drugs possess the potential to mitigate aging-related pathological conditions [57].

The emerging multifaceted roles of Shh signaling under various neurological conditions are intriguing and the underlying mechanisms are worth further exploring. The role of Shh in mature neural tissue may provide therapeutic possibility, especially in devastating neurodegenerative disorders. In this article, we will focus on the potential action of Shh signaling in neurological diseases that may involve neurogenesis, anti-oxidation, anti-inflammation, and autophagy.

## 2. Sonic Hedgehog and Neurogenesis in Neurological Diseases

In the adult brain, neurogenesis continues in two regions: the subventricular zone (SVZ), which lines the lateral ventricles and gives rise to new interneurons that reach the olfactory bulb via the rostral migratory stream (RMS), and the subgranular zone (SGZ) of the dentate gyrus (DG) in hippocampus, which generates new granule cells [58]. Neurogenesis is modulated by both physiological stimuli, such as aging factor, exercise, learning, and genetic background [59,60,61,62] as well as pathophysiological conditions, such as seizure [63,64,65] and cerebral ischemia [66,67,68]. Manipulations, especially enhancement, of neurogenesis may carry therapeutic potential that can be applied in brain dysfunction with neuronal injury, such as cerebrovascular diseases and other neurodegenerative disorders.

Neurogenesis is important for neural tissues to renew, replace, and repair, thereby contributing to the maintenance of proper functions. The well-known regulators of neurogenesis in SVZ and SGZ include Wnt, bone morphogenetic protein (BMP), and Shh [69,70]. Several lines of evidence have revealed the crucial role of Shh in neurological diseases concerning the capability of neurogenesis [30,32,71] and, in this article, we will focus on Shh in neurogenesis as well.

Various compounds and drugs capable of modulating Shh pathway and neurogenesis have been tested. It was reported that salvianolic acid, an antioxidant and a free radical scavenger, promoted functional recovery and neurogenesis via activation of Shh after stroke in mice. It enhanced proliferation of NPCs and elevated long-term survival of newborn neurons in the SVZ; it also increased the expression of Shh and Ptch along with heightened nuclear translocation of Gli1 in the peri-infarct region, thereby causing robust production of BDNF and nerve growth factor. Notably, the Smo inhibitor cyclopamine markedly attenuated the beneficial outcomes of salvianolic acids [72], suggesting that Smo plays a crucial role in the observed protective mechanisms of salvianolic acids.

Resveratrol, a polyphenol derived from grapes, is known to have neuroprotective effects against ischemic stroke in the brains via various mechanisms such as anti-oxidation, anti-inflammation, and anti-apoptosis [73,74,75]. It was found that resveratrol considerably increased expression of Shh, Ptch-1, Smo, and Gli-1 at mRNA levels; furthermore, resveratrol also enhanced nuclear translocation of Gli-1. Inhibition of the Shh signaling pathway with the Smo inhibitor cyclopamine completely reversed the effects of resveratrol. These results suggest that resveratrol decreased cerebral ischemic injury and improved neurological function by upregulating Shh signaling pathway [76].

Epidemiologic evidence suggests that consumption of green tea is associated with reduced mortality of cardiovascular disease, inflammatory diseases, diabetes, and stroke as well as prevention and treatment of cancer [77,78]. Epigallocatechin-3-gallate (EGCG) is the major polyphenol and an active ingredient in green tea. It was shown that EGCG treatment significantly enhanced neurogenesis based on the increased numbers of 5-bromo-2′-deoxyuridine (BrdU)-labeled cells in the dentate gyrus of adult mice as well as in adult hippocampal NPCs. EGCG also triggered a vigorous upregulation of Shh signaling, namely expression of Ptch at mRNA and protein levels, as well as enhanced Gli expression in cultured NPCs; moreover, blockade of the Shh signaling attenuated EGCG-induced hippocampal neurogenesis [79].

In addition to those compounds derived from nature products like resveratrol and EGCG, small molecules capable of directly activating Shh signaling cascades have also been shown to enhance neurogenesis. For example, SAG, abbreviation for Smoothened AGonist, was isolated from the screening of 140,000 synthetic compounds using a Gli-luciferase reporter assay in mouse C3H10T1/2 cells [80,81]. Being a chlorobenzothiophene-containing Hh pathway agonist, SAG binds to the Smo heptahelical bundle in a manner similar to the Smo inhibitor cyclopamine [80]. In one study, SAG was administered 3–7 days after ischemic stroke in mice to enhance survival of newborn NSCs derived from both SVZ and SGZ in the ischemic brains. After one month of stroke, both cognitive function and locomotor activity were significantly improved in the SAG group compared to the vehicle group. These data validate a critical role of Shh pathway in post-stroke brain restoration and functional improvement. These results also indicate that modulation of Shh pathway can prolong treatment time window and could be a potential treatment strategy for ischemic stroke [82].

Neurogenesis impairment is considered a main determining factor of the intellectual incompetence observed in patients with Down Syndrome (DS), a genetic pathology caused by triplication of human chromosome 21 [83,84]. In a Ts65Dn mouse model of DS, it was demonstrated that the amyloid precursor protein (APP) triplicated gene impairs proliferation of NPCs from SVZ in the hippocampus [85,86] because high levels of the intracellular domain from APP cleavage by γ-secretase can raise the transcription of Ptch1, which is known to repress Shh pathway [85,86]. ELND006 is an inhibitor of γ-secretase that restores the Shh pathway and fully recovers the impaired neurogenesis in Ts65Dn pups [87]. In another study, it was demonstrated that early inhibition of γ-secretase can improve brain development in DS [88]. These findings denote the potential therapeutic application of Shh in DS. Consistently, the Smo agonist SAG corrects the structural and cognitive deficits in the Ts65Dn mice; intriguingly, a single treatment of newborn mice with SAG results in normal cerebellar morphology, behavioral improvement in the Morris water maze task, and partial rescues of n-methyl-d-aspartate (NMDA) receptor-dependent synaptic plasticity in adults [89]. These findings suggest that Shh agonists may carry therapeutic potential in the treatment of DS via enhancement of neurogenesis.

Cerebrolysin, a peptide preparation with various neurotrophic factors, can augment neurogenesis with better functional outcome in ischemic stroke and neurodegenerative diseases [90,91]. It was demonstrated that cyclopamine, which inhibits Smo in the Shh pathway, can fully reverse the beneficial actions of cerebrolysin on functional recovery of neurological deficits in the in vivo ischemic model [50]. Intrathecal delivery of recombinant Shh protein to the animals subject to ischemic stroke improved behavioral and functional recovery, which may be related to its effects on neurogenesis in the SVZ [92]. These results once again revealed the importance of Shh signaling pathway in enhancing neurogenesis under various neurological conditions, including cerebral ischemia.

Electroconvulsive seizure (ECS), a neuromodulatory modality to treat major depressive disorder [93], can enhance hippocampal neurogenesis, mossy fiber sprouting, synaptic reorganization, and neural plasticity in the adult brain [94,95,96]. It was demonstrated that ECS increases proliferation of adult hippocampal progenitors and these effects were fully blocked by cyclopamine, the pharmacological inhibitor of Shh signaling. These results suggest that the Shh pathway may be a critical mechanism for ECS to enhance adult hippocampal neurogenesis [97].

Enhancing endogenous stem cells and promoting regeneration of the injured nervous system may be vital approaches in patients suffering from brain injury. In an in vitro organotypic stretch injury model, it was shown that endogenous glial fibrillary acidic protein (GFAP)-positive NSCs/NPCs in the postnatal mouse cortex are activated following a stretch injury equivalent to a severe traumatic brain injury (TBI); intriguingly, these cells are likely to arise from the cortical parenchyma but not from the SVZ. More importantly, upregulation of Shh signaling following TBI was observed. Given the correlative evidence linking restoration of regenerative potential to upregulation of Shh pathway, these findings suggest a possibility of using this endogenous source of GFAP-positive stem cells for repair following TBI, in which Shh plays a key role in regulating their proliferation [98].

Reduction of neurogenesis in the brain is one of the main causes of dementia in AD and, on the contrary, modifying the course of hippocampal neurogenesis assists patients with AD [99]. It was shown that a massive shortfall in Ptch1 and Gli1 was observed in the hippocampus in the aged AD transgenic mice that would compromise the ability of genesis in both NSCs and glial precursor cells, although contents of these two proteins were substantially higher at young ages. The comparable findings in autopsied AD brains confirmed this discovery in the mouse model [100]. These observations suggest that deregulation of Ptch1-Gli1 signaling may result in abnormal loss of NSCs and glial precursor cells, thus contributing to cognitive decline in AD brains.

Administration of kainic acid (KA) into rodents, which results in hippocampal damage, neuronal death, and seizures, is a well-characterized model to study human neurodegeneration [101,102]. It was shown that KA induces hippocampal neuronal death along with activation of microglia and astrocytes. The mitogen Shh is upregulated in reactive astrocytes in response to the insults and modulates astrocyte activation and proliferation. The activated Shh-Gli pathway in various glial cells is responsible for proliferation in post-neurodegenerative lesions [39].

Various approaches may be applied to enhance neurogenesis under different neurological conditions. Umbilical cord blood mononuclear cells (UCBMC) can alleviate brain damage [103,104] and promote the proliferation of endogenous NSCs [105]. UCBMC, delivered at 24 h after hypoxia/ischemia (HI) in neonatal rats, can advance neuronal differentiation and decrease glial differentiation in the cerebral cortex via the Hh signaling pathway [106].

Overall, understanding how Shh signaling is affected under various neurological conditions and precise control of the Shh signaling, and hence the capability of neurogenesis, is crucial in future clinical application of Shh pathway in those neurodegenerative disorders in which neurogenesis plays a pivotal role.

## 3. Sonic Hedgehog and Antioxidation in Neurological Diseases

It is well known that living cells can produce excessive reactive oxygen species (ROS) under various stimuli such as hypoxia, serum deprivation, and cytokine stimulation [107,108]. Multiple sources of ROS exist that include NADPH oxidase, 5-lipoxygenase, and mitochondria [109]. Among them, mitochondria are the major organelle that produces ROS within cells [110,111,112]. During aerobic respiration, free electrons on the mitochondria may leak out from electron transport chain to react with molecular oxygen, thus producing superoxide anion as metabolic byproducts. Nitric oxide (NO) can react with superoxide anion to generate the highly reactive peroxynitrite anion (ONOO^−^) that modifies and damages DNA, proteins, and lipids. Collectively, modification of these cellular macromolecules by ROS and/or reactive nitrogen species (RNS) plays an important role in a number of different physiological/pathological conditions, particularly aging, cancer, ischemia-reperfusion injury, and chronic neurodegeneration [113,114,115,116]. Counteracting the formation of excessive ROS via endogenous antioxidative mechanisms is critical for cells to survive and decreasing the ROS formation should have positive impacts in treating ROS-related disorders, including cerebral ischemia and neurodegenerative diseases. Recent studies have indicated that the Shh signaling pathway is involved in these diseases, but the underlying mechanisms remain to be clarified in terms of counteracting excessive ROS production [32].

It was shown that exposure of primary cortical neurons to hydrogen peroxide (H_2_O_2_) decreased cell viability and inhibition of endogenous Shh signaling further aggravated the detrimental effect of H_2_O_2_ in neurons. Exogenous Shh increased the activities of glutathione peroxidase (GSH-PX) and superoxide dismutase (SOD), attenuated malondialdehyde (MDA) formation, promoted the expression of anti-apoptotic Bcl-2, and suppressed expression of pro-apoptotic Bax in H_2_O_2_-treated neurons. Expression of two neurotrophic factors, namely vascular endothelial growth factor (VEGF) and BDNF, was increased with Shh activation. These findings reveal that enhancement of Shh signaling can protect cortical neurons against oxidative damage and apoptosis, thus denoting a potential role of Shh for the therapeutic effects in brain ischemia and other neurodegenerative disorders [117]. It was also reported that exogenous Shh could augment the expression of p-Akt and diminish the activity of p-ERK, suggesting that Shh/PI3K/Bcl-2 pathway may be involved in the protection of neurons against H_2_O_2_-induced oxidative stress and apoptosis [118]. In a similar in vitro model, it was demonstrated that Shh, acting as a prosurvival factor, plays a crucial part in neurite outgrowth in the H_2_O_2_-treated cortical neurons. Potential mechanisms underlying the antioxidative efficacies of Shh may include counteracting ROS release, prevention of mitochondrial dysfunction, promotion of ATP production, as well as preservation of mitochondrial complex II activities against oxidative stress [119].

It was shown that subarachnoid hemorrhage (SAH) increased mRNA and protein levels of Shh, Ptch1, and Gli-1 in the cerebral cortex. Cyclopamine, inhibitor of Smo in the Shh pathway, augmented the MDA formation and reduced the enzyme activities of SOD and GSH-Px in the brain. These results suggest that Shh pathway may play a protective role in SAH, notably by preventing oxidative stress in cortex via activation of antioxidative and detoxifying enzymes [120]. In another study, purmorphamine (PUR), another agonist of Smo in addition to SAG, enhanced the expression of Shh and Gli that was decreased by SAH while attenuating SAH-dependent induction of Ptch and resulted in protection; all of these PUR actions were blocked by cyclopamine. Mechanistically, PUR treatment markedly decreased MDA content that was accompanied by the heightened expression of nuclear factor erythroid 2-related factor 2 (Nrf2) and its target gene heme oxygenase-1 (HO-1). Thus, the effect of PUR against SAH-induced injury in rats may be mediated in part by anti-apoptotic and antioxidative mechanisms, increased pERK levels, and enhanced Shh signaling in the frontal cortex [121].

Gli1 and Ptch1 are critical effectors in the Shh pathway; indeed, both of them are also transcriptional targets downstream of Shh signaling. In a rat model of cerebral ischemia, inhibition of Shh pathway led to decreased expression of Gli1, Ptch1, and SOD1 in ischemia-affected brain tissue accompanied by increased brain water content, infarct volume, and behavioral deficits. All these results imply that reduction of Shh signaling pathway aggravates ischemic injury in rats that is associated with down-regulation of Gli1, Ptch1, and SOD1 [36].

The underlying mechanisms of autism spectrum disorders (ASD) are still not clear [122]; however, as with other neurodegenerative diseases, oxidative stress may play a pathological role in this brain disorder [123]. It was demonstrated that autistic children had a notably higher level of ROS; further, heightened serum contents of Shh protein were detected in the children of autism, whereas BDNF content was considerably reduced in mild, but not severe, form of autistic children. This study demonstrates a correlation among Shh, BDNF, and ROS in autistic children and suggests a critical role of oxidative stress and Shh in ASD [124].

Oxidized low-density lipoprotein (oxLDL) increases the expression of pro-inflammatory genes, resulting in monocyte recruitment into the vessel wall with dysfunction of vascular endothelial cells [125]. oxLDL is elevated under several neurological conditions and induces disruption of blood-brain barrier (BBB) with resultant formation of cerebral edema [126]. Treatment of murine brain microvascular endothelial cells (MBMECs) with oxLDL increased intracellular ROS and MDA formation, decreased NO release, and reduced cell viability. These effects also involve the Shh signaling. This is because the mRNA and protein levels of Shh, Smo, and Gli1 were all considerably decreased after incubation with oxLDL, while overexpression of Shh diminished oxLDL-induced elevation of permeability in MBMECs. These results may denote the potential role of Shh pathway in BBB dysfunction with therapeutic implication in various neurological disorders [127,128].

Despite limited studies implicating Shh signaling pathway in neurological disorders, especially in neurodegenerative diseases, emerging evidence revealed that this pathway may play an important role in counteracting oxidative stress. Additional evidence also shows that Shh may act like an immediate early gene that is expressed quickly in response to acute insults to exert its beneficial effects [117,129,130] with activation of the endogenous neuroprotective mechanisms. More studies are needed to support this notion and develop an efficacious therapeutic regimen in this regard especially in neurodegenerative diseases.

## 4. Sonic Hedgehog and Anti-Inflammation in Neurological Diseases

It is well-known that inflammation may play a crucial role in the pathogenesis of neurodegenerative diseases such as AD, amyotrophic lateral sclerosis (ALS), Parkinson’s disease (PD), and multiple sclerosis (MS). Despite various etiologies such as genetic mutations, infections, misfolded proteins, and brain injury, neuronal damages often involve both adaptive and innate immune systems at various stages of disease in the CNS [131,132]. Inflammation-related signaling pathways in neurological disorders includes the Toll-like receptors pathway, the mitogen-activated protein kinases pathway, and the nuclear factor-kappa B pathway. In particular, mutual interaction has been noted in inflammation and oxidative stress. For instance, superoxide anion from NADPH oxidase in activated microglia may interact with NO produced during inflammatory responses to form the active oxidant peroxynitrite [133]. Given that these responses may have direct impacts on disease progression, they could serve as the targets for therapeutic intervention.

Evidence showed that Shh signaling pathway plays a role in coping with ROS overproduction under conditions of neurological diseases [36,117,121] and is also possibly involved in inflammatory reactions. It was reported that Shh pathway is activated by acute brain injury and regulated by injury-related inflammation [37]. Shh pathway is intensively induced three days after brain injury and returns to baseline condition by 14 days. Shh expression correlates with Gli activation and is confined to those reactive astrocytes with GFAP expression. Blockade of Shh pathway by cyclopamine decreases Gli expression and attenuates considerably the extents of proliferation with reduction in the numbers of reactive astrocytes in the injured cortex [37].

In KA-induced neurodegeneration, the Shh expression is increased in reactive astrocytes. The peak activity of Shh was detected at seven days along with increased Gli activity and heightened proliferation in several types of glial cells. Thus, the Shh/Gli pathway is activated and leads to proliferation of reactive glial cells in response to KA-induced lesions [39].

The BBB that is composed of astrocytes, capillary endothelial cells, and pericytes is important for CNS homeostasis. The BBB function is altered in a number of neurological diseases like AD, PD, ALS, stroke, epilepsy, and brain trauma [134,135]. Several studies revealed that compromised integrity of BBB or its dysfunction may involve Shh pathway during inflammation [136,137,138,139]. Shh released from astrocytes plays an important role in the maintenance of BBB integrity [140]. Through diminishing expression of proinflammatory mediators and adhesion/migration of leukocytes, Shh can promote the quiescence of immune response in endothelial cells of BBB both in vivo and in vitro [140]. Further, it was shown that interleukin-1β (IL-1β) induces BBB disruption by downregulating Shh expression in astrocytes. Enhancing the expression of astrocytic Shh may carry a therapeutic potential to restore disrupted BBB in patients with various neurological diseases [141].

Wip1, a nuclear phosphatase that can be activated under various types of stresses, is involved in aging, neuroinflammation, neurogenesis, and tumorigenesis [142,143,144,145,146]. A cross-talk between Wip1 pathways and Shh may accelerate tumorigenesis [147]. Wip1 can regulate lipopolysaccharide (LPS)-induced BBB dysfunction and neuroinflammation through Shh signaling pathway. Silencing of Wip1 can increase inflammatory cytokines such as IL-1β, IL-6, IL-12, and tumor necrosis factor-α (TNF-α) of the BBB induced by LPS, while overexpression of Wip1 results in an opposite effect in the expression of these cytokines and decreases the extent of inflammatory response. Consistent with the above findings, Wip1 overexpression increases Shh signaling and silencing of Wip1 represses Shh. These results indicate that Wip1 may counteract LPS-induced inflammatory response and BBB disruption while maintaining the BBB integrity via augmentation of Shh signaling [137].

BBB dysfunction may involve increased matrix metalloproteinase-9 (MMP-9) activity and breakdown of tight junction protein (TJP) [136,148,149]. CNS tuberculosis has a high mortality and morbidity associated with severe inflammation and BBB dysfunction [150,151]. In a co-culture model to study BBB integrity, which includes endothelial cells and astrocytes with conditioned medium from *Mycobacterium tuberculosis* (Mtb)-infected monocytes, expression of TJP is regulated by Shh via transcription factor Gli-1. The breakdown of TJP is related to secretion of MMP-9. SAG, exogenous Shh, or knockdown of MMP-9 expression can decrease BBB permeability and increase the expression of TJP in the Mtb-stimulated co-cultures [136]. These findings denote the relevance of Shh pathway in BBB integrity in CNS inflammation and infection.

Human immunodeficiency virus type-1 (HIV)-associated neurocognitive disorder (HAND) includes asymptomatic neurocognitive impairment, mild neurocognitive disorder, and HIV-associated dementia [152]. The activated/infected leukocytes are recruited into CNS through disrupted BBB due to persistent neuroinflammatory conditions. Using a rodent model of HAND, administration of SAG restored BBB integrity and also ameliorated the neuropathological deficits in infected mice. These results suggest that Shh signaling carries a therapeutic potential in HAND [138,139].

Several examples are presented here to show the pivotal role of Shh signaling pathway in inflammation-related neurological diseases. Potential use of SAG or Shh mimetic to counteract the inflammatory status offers a therapeutic option in term of mutual reaction between oxidative stress and inflammation; both are significant contributors to various neurodegenerative diseases. Additional future studies will provide evidence to support the beneficial effect of these Shh-related compounds for future clinical use.

## 5. Sonic Hedgehog and Autophagy in Neurological Diseases

Autophagy, a highly regulated process that breaks down organelles and macromolecules through lysosomal degradation, is essential for maintenance of intracellular homeostasis while the cell is under starvation, differentiation, and normal growth control [153,154]. Autophagy may function as a pro-survival mechanism throughout the period of nutrient shortage when cytoplasmic contents are reprocessed for ATP generation and production of nascent macromolecules. The role of autophagy in neurodegenerative diseases is just beginning to be elucidated [155,156,157]. Various insults during neurodegeneration can cause oxidative stress that damages multiple intracellular molecules. Thus, effective clearance of damaged organelles to break down the macromolecules for generation of building blocks, such as amino acids and nucleotides, for salvage would be protective under starvation conditions. Furthermore, removal of damaged organelles may also prevent apoptosis, especially when mitochondrial integrity is compromised. Through collaboration with ubiquitin-proteasome system [158], the protective role of autophagy in neurodegenerative process may be attributable to its ability to clear protein aggregates and damaged cytoplasmic organelles [159]. In contrast, defective autophagy may contribute to the pathogenesis of aging and neurodegenerative diseases [160]. However, uncontrollable autophagy would lead to aggressive digestion of affected neurons leading ultimately to neuronal death [161,162].

The induction of autophagy has been shown in mouse cortex and striatum after various brain insults including ischemia or during progression of various neurodegenerative diseases such as AD, PD, and HD [163,164,165,166,167]. The microtubule-associated protein 1 light chain 3 (LC-3) is a marker protein for autophagy as it is required for autophagosome formation via its conversion from cytosolic LC3-I to membrane-bound LC3-II [168]. Genetic deletion of essential autophagy genes Atg 5 and 7 in mice results in neurodegeneration suggesting that autophagy is vital for normal neuronal function [169]. Nevertheless, autophagy could be a double-edged sword: it is protective in response to mild stress, but it could also be stressful and detrimental to neuronal survival as a result of over-activation by a more severe stress such as serious ischemia [170]. Delineation of the role of autophagy in various neurological conditions may broaden our knowledge and enhance our ability to manipulate the potential cell survival or death pathways and mitigate the neuronal injury.

Using the autophagy marker LC-3 by immunoblot analysis and immunocytochemistry, it was reported that autophagy pathway in Shh-exposed neurons was activated. Autophagosomes and various associated morphological changes were found in synaptic terminals in Shh-exposed neurons and the Shh-induced autophagy was dependent on class III Phosphatidylinositol 3-kinase complexes (PtdIns3K) [171]. However, whether Shh signaling can protect neurons against dysfunction and degeneration in AD or other neurodegenerative disorders still awaits elucidation. Evidence revealed the connection between Shh and autophagy because the Hh signaling pathway reduced the formation of autophagosome, both under basal as well as autophagy-induced conditions. The potential implications of Shh in autophagy is the ability to control protein homeostasis under physiological and pathological conditions [172]. Despite limited studies concerning Shh and autophagy reported in neural cells [171], numerous previous studies referring to Shh and autophagy in the disorders of other systems should inspire future investigation of Shh in relation to autophagy under normal as well as diseased neurological conditions [173,174,175,176].

In the intestinal epithelial cells derived from the Shh conditional knockout mice, it was shown that loss of Shh can alter ileal secretory cell maturation with endoplasmic reticulum stress and an evident reduction in autophagy [175]. Dysregulation of miRNAs, a contributing factor to autophagy, is implicated in a range of pathological conditions, including hepatic fibrosis. Among them, miR-148a interacts with the 3′-untranslated regions (3′-UTRs) of growth arrest-specific gene 1 (Gas1) transcripts to inhibit its expression. Gas1 encodes a surface binding receptor for Hh and assists the Shh signaling pathway in reducing autophagosome formation and thus have the potential to serve as a target for future development of novel therapeutic strategies against related diseases [173].

Shh pathway has been reported to protect cardiomyocytes in myocardial infarction (MI), but the underlying mechanism is not well defined [177,178]. It was revealed that Shh triggers AMPK-dependent autophagy in cardiomyocytes under oxygen glucose deprivation (OGD), a model mimicking the ischemic condition. SAG, the Shh pathway agonist, increased the expression of LC3-II and caused the formation of autophagosomes. These results indicate an important function of autophagy in Shh-induced cellular protection [174].

Autophagy in vascular smooth muscle cells (SMCs) is known to increase plaque stability [179,180]. Shh is expressed in atherosclerotic lesions and stimulates proliferation of vascular SMCs. It was demonstrated that both LC3-II and Shh protein expression were augmented within SMCs of neointimal lesions in the common carotid artery of mouse. Overexpression of mouse Shh in vascular SMCs increased the levels of LC3-II and also stimulated AKT phosphorylation. Shh-induced autophagy was further confirmed by the formation of autophagosomes as detected by immunostaining and electron microscopy, which was inhibited by AKT inhibitor IV. Shh promoted SMC proliferation, which was impeded not only by AKT inhibitor IV but also by cyclopamine. These findings suggest that Shh enhances autophagy of vascular SMCs by engaging AKT activation, thus indicating a crucial role of autophagy in Shh-induced cellular effects [176].

In the future studies of patients with neurodegenerative diseases or animal models of neurological disorders, manipulation of Shh signaling and alteration of autophagy by pharmacological or molecular approaches will improve our understanding towards these diseases and may guide the way to novel methods for their prevention and treatment.

## 6. Conclusions and Future Perspectives

**Figure 2:**
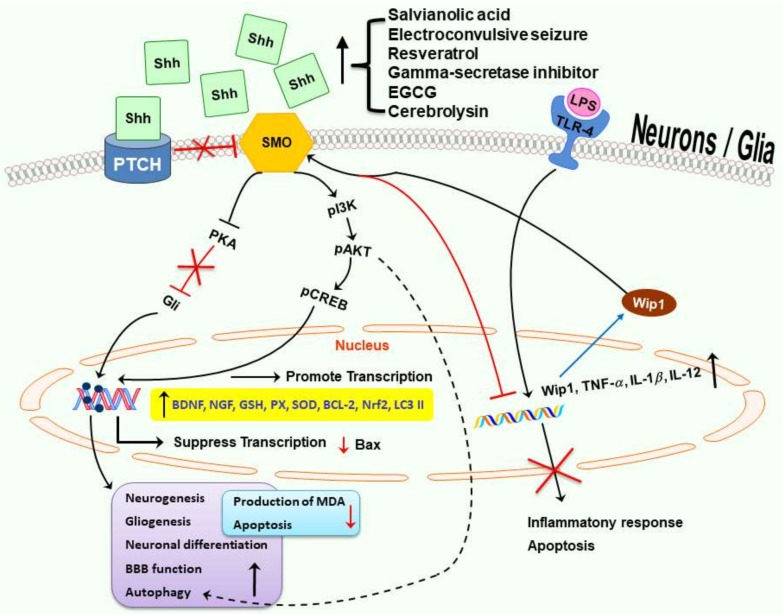
Multiple potential neuroprotective mechanisms in Shh signaling pathway. In the presence of Shh, inactivated Ptch relieves its suppression on Smo to activate several downstream pathways. The neuroprotective mechanisms of Shh may involve enhancement of neurogenesis, gliogenesis, autophagy, mitochondrial function, BBB function, anti-oxidation, anti-inflammation, and anti-apoptosis. Black solid or dashed arrows indicate promotion or increasing; red lines with an “T” and/or “X” indicate inhibition, suppression, or decreasing; blue arrows indicate translation.

Shh, originally revealed as a mitogen and crucial for development including CNS patterning and polarity, is now appreciated for its various functions to cope with different types of stresses. These include mitigation of oxidative stress and inflammation as well as modulation of autophagy-related mechanisms to help our body adjust to environmental challenge. In addition, Shh pathway also improves mitochondrial function and carries an antiaging effect. The potential protective mechanisms of Shh in adult neurological disorders are shown in Figure 2. More studies are needed to support the crucial roles of Shh signaling pathway in adult neurological disease, especially in neurodegenerative diseases currently without effective treatment, such as AD, PD and HD. Small molecules or compounds capable of activating Shh pathway may have the potential to treat or delay the progression of these devastating neurological disorders.

## Abbreviations

3-NP	3-nitropropionic acid
AD	Alzheimer’s disease
ALS	amyotrophic lateral sclerosis
APP	amyloid precursor protein
ASD	autism spectrum disorders
BBB	blood-brain barrier
BDNF	brain-derived neurotrophic factor
BMP	bone morphogenetic protein
BrdU	5-bromo-2′-deoxyuridine
CK1	casein kinase 1
CNS	central nervous system
DG	dentate gyrus
DS	Down syndrome
ECS	electroconvulsive seizure
EGCG	epigallocatechin-3-gallate
EPO	erythropoietin
FU	Fused
Gas1	growth arrest-specific gene 1
Gli	glioma-associated oncogene homolog
GSK3	glycogen synthase kinase-3
GSH-PX	glutathione peroxidase
H_2_O_2_	hydrogen peroxide
HAND	human immunodeficiency virus type-1 (HIV)-associated neurocognitive disorder
HD	Huntington’s disease
Hh	hedgehog gene
HI	hypoxia ischemia
IL-1β	interleukin-1β
KA	kainic acid
LC-3	microtubule-associated protein 1 light chain-3
LPS	lipopolysaccharide
MBMECs	murine brain microvascular endothelial cells
MCAO	middle cerebral artery occlusion
MDA	malondialdehyde
MMP-9	matrix metalloproteinase-9
MS	multiple sclerosis
Mtb	mycobacterium tuberculosis
NO	nitric oxide
NPCs	neural progenitor cells
NSCs	neural stem cells
OGD	oxygen glucose deprivation
PD	Parkinson’s disease
ONOO^−^	peroxynitrite anion
oxLDL	oxidized low-density lipoprotein
PD	Parkinson’s disease
PKA	protein kinase A
Ptch	Patched
PUR	purmorphamine
RMS	rostral migratory stream
RNS	reactive nitrogen species
ROS	reactive oxygen species
SAG	smoothened agonist
SAH	subarachnoid hemorrhage
SGZ	subgranular zone
Shh	sonic hedgehog
Shh-N	N-terminal fragment of Shh
SMCs	smooth muscle cells
Smo	smoothened
SODs	superoxide dismutases
SUFU	suppressor of FU
SVZ	subventricular zone
TBI	traumatic brain injury
TJP	tight junction protein
TNF-α	tumor necrosis factor-α
UCBMC	umbilical cord blood mononuclear cells
VEGF	vascular endothelial growth factor
